# Mapping European research networks providing health data: results from the InfAct Joint Action on health information

**DOI:** 10.1186/s13690-021-00766-2

**Published:** 2022-01-10

**Authors:** Brigid Unim, Elsi Haverinen, Eugenio Mattei, Flavia Carle, Andrea Faragalli, Rosaria Gesuita, Martin Thissen, Linda Abboud, Tiziana Grisetti, Petronille Bogaert, Luigi Palmieri

**Affiliations:** 1 Department of Cardiovascular, Endocrine-metabolic Diseases and Aging, Istituto Superiore di Sanità, Rome, Italy; 2 Department of Public Health and Welfare, Finnish Institute for Health and Welfare (THL), Helsinki, Finland; 3 Center of Epidemiology, Biostatistics and Medical Information, Marche Polytechnic University, Ancona, Italy; 4 Department of Epidemiology and Health Monitoring, Robert Koch Institute, Berlin, Germany; 5 Epidemiology and Public Health, Sciensano, Brussels, Belgium

**Keywords:** Research network, Health information, Data collection, Quality assessment, Data availability, Data accessibility

## Abstract

**Background:**

Research networks offer multidisciplinary expertise and promote information exchange between researchers across Europe. They are essential for the European Union’s (EU) health information system as providers of health information and data. The aim of this mapping exercise was to identify and analyze EU research networks in terms of health data collection methods, quality assessment, availability and accessibility procedures.

**Methods:**

A web-based search was performed to identify EU research networks that are not part of international organizations (e.g., WHO-Europe, OECD) and are involved in collection of data for health monitoring or health system performance assessment. General characteristics of the research networks (e.g., data sources, representativeness), quality assessment procedures, availability and accessibility of health data were collected through an ad hoc extraction form.

**Results:**

Fifty-seven research networks, representative at national, international or regional level, were identified. In these networks, data are mainly collected through administrative sources, health surveys and cohort studies. Over 70% of networks provide information on quality assessment of their data collection procedures. Most networks share macrodata through articles and reports, while microdata are available from ten networks. A request for data access is required by 14 networks, of which three apply a financial charge. Few networks share data with other research networks (8/49) or specify the metadata-reporting standards used for data description (9/49).

**Conclusions:**

Improving health information and availability of high quality data is a priority in Europe. Research networks could play a major role in tackling health data and information inequalities by enhancing quality, availability, and accessibility of health data and data sharing across European networks.

**Supplementary Information:**

The online version contains supplementary material available at 10.1186/s13690-021-00766-2.

## Background

Research networks (RNs) offer multidisciplinary expertise and promote information exchange between researchers across Europe and extra-European countries. They are essential for health information systems as providers of health information and data, collaborating with various sectors, such as government, industry, academia and independent research groups. Many RNs include in their activities the collection of data and information from multiple verified sources into compiled databases, following standardized procedures. Therefore, their data and information could be considered more reliable compared to those from individual sources. Among the advantages of RNs are data collection and sharing, collaborative research across different geographical areas, strengthening research capacities and quality by conducting research according to standardized methods and practices, and provision of training courses for network members and the wider community. Ultimately, collaboration in RNs enhances the productivity of individual researchers [1, 2].

There are various definitions of a network and types of RNs. A network is generally a relationship between three or more individuals or groups characterized by shared objectives to achieve common goals [2]. Networks could be informal, like social media platforms (e.g., ResearchGate, LinkedIn) that facilitate informal networking among researchers who can exchange research materials and information about job opportunities with each other. A formal RN is usually funded for a specific purpose and timeframe, has an administrative structure and rules to coordinate the activities of network members that could be institutions or organizations with common goals (e.g., Better Statistics for Better Health for Mothers and their Newborns in Europe-Euro-Peristat, European Cardiovascular Indicators Surveillance Set-EUROCISS, Survey of Health, Ageing and Retirement in Europe-SHARE, European Collaboration for Healthcare Optimization-ECHO, Multinational MONItoring of Trends and Determinants in CArdiovascular Disease-MONICA). According to their goals and funding availability, RNs may also differ in size and structure ranging from selected members from few organizations to numerous members from institutions across the globe. RNs may also differ in lifespan, which depends on financial resources, research relevance, and multidisciplinary collaborations [2]; these factors may influence the network’s capacity in achieving their objectives and goals. Given that the activities of a network are time limited and the development of evidence-based recommendations and their translation into policy and practice may require a longer time frame, the lifespan of RNs may cause fragmentation of health research worldwide [3].

The present study is part of the Joint Action (JA) on Health Information InfAct (Information for Action), that was launched in 2018 and will be active until 2021. The JA involves 40 partners from 28 EU and 4 associated countries working together towards a sustainable infrastructure for the European Union’s (EU) health information to support evidence-based policy and research activities. The purpose of this mapping exercise is to identify and evaluate EU RNs that are not part of international organizations (e.g., World Health Organization-WHO Regional Office for Europe, Organisation for Economic Co-operation and Development-OECD, European Statistical Office-Eurostat) and are involved in collection of data for health monitoring or health system performance assessment.

## Methods

For the purpose of this study, a RN is defined as a project involving at least two institutions or stakeholders in a country (national RN) or institutions/stakeholders in at least two countries (international RN). RNs were retrieved through a web-based desk research between April and June 2019. The desk search was conducted using publicly available information on the European Commission’s Community Research and Development Information Service (CORDIS) database and on the websites of international organizations (i.e., WHO-Europe, Eurostat, OECD, European Centre for Disease Prevention and Control-ECDC, European Food Safety Authority-EFSA, European Monitoring Centre for Drugs and Drug Addiction-EMCDDA). A Google search was also performed using the terms *European research network(s) AND health information*, and the first 10 pages were analysed. Additional RNs were identified through a cross-sectional study addressing InfAct project partners on health data collection methods and procedures across EU Member States (EU MS). The study led to the identification of EU research projects, which were also part of EU RNs. EU RNs that are not part of international organizations (e.g., WHO-Europe, OECD) and are involved in data collection for health monitoring or health system performance assessment are included in the present study. The websites of the identified networks were then evaluated according to the following sections of an ad hoc extraction form:

i) General characteristics (i.e., name and acronym of the RN; responsible authority and funder; years of activity; main objectives; principal area of research; coordinating and participating countries; level of representativeness; types of data sources used; data sharing activities; main diseases, health topics or risk factors considered; elaboration of indicators);
ii) Quality assurance (i.e., information on data quality assessment);
iii) Data availability (i.e., availability of micro or macrodata, data formats, and metadata standards);
iv) Data accessibility (i.e., criteria for exchange and sharing of statistical data and metadata).

Regarding quality assurance, the RNs were assessed by four researchers independently through 10 quality dimensions or criteria (Table 1), of which eight were defined by Eurostat [4] (i.e., relevance, accuracy, timeliness, punctuality, comparability, coherence, accessibility and clarity) and two by ECHO (coverage and internal reliability) [5].

**Table 1:** Quality criteria used to assess information provided by research networks on their health data

Quality criteria	Definition
**Relevance**	Is the degree to which statistics meet current and potential user needs. It refers to whether all statistics that are needed are produced and the extent to which concepts (definitions, classifications etc.) reflect users’ needs.
**Accuracy**	Statistically, it denotes the closeness of computations or estimates to the (unknown) exact or true values.
**Timeliness of information**	reflects the length of time between its availability and the event or phenomenon it describes.
**Punctuality**	It refers to the time lag between the release date of data and the target date when it should have been delivered, for instance, with reference to dates announced in some official release calendar, laid down by regulations or previously agreed among partners
**Comparability**	Aims at measuring the impact of differences in applied statistical concepts and measurement tools/procedures when statistics are compared between geographical areas, non-geographical domains, or over time.
**Coherence**	It is the adequacy of statistics data to be reliably combined in different ways and for various uses. When originating from different sources, and in particular from statistical surveys of different nature and/or frequencies, statistics may not be completely coherent in the sense that they may be based on different approaches, classifications and methodological standards.
**Accessibility**	Refers to the physical conditions under which users can obtain data: where to go, are access to data free or restrictive, etc.
**Clarity**	Refers to the data’s information environment whether data are accompanied with appropriate documentation and metadata, illustrations such as graphs and maps, whether information on their quality is also available (including limitation in use etc.) and the extent to which additional assistance is provided.
**Coverage**	Measures the extent to which the sample stored describes actual performance. Also represents a measure of the potential relevance of the data stored.
**Internal reliability**	A measure of whether the information stored is consistent over the years. It is a necessary condition for accurate estimations.

## Results

### General characteristics of the networks

A total of 57 RNs (Table 2) were identified and, to date, eight RNs are still active: Euro-Peristat, Committee of Nordic Assisted Reproductive Technology and Safety-CoNARTaS, EUropean Best Information through Regional Outcomes in Diabetes-EUBIROD, European Health Examination Survey-EHES, Extracorporeal life support association-ELSO, Research on Children and Adults Born Preterm-RECAP preterm, SHARE, and Commonwealth Fund Multinational Comparisons of Health Systems Data-MultiCom.

**Table 2:** European research networks involved in health data collection

Research network	Acronym	Years of activity
Best Information through Regional Outcomes: a Shared European Diabetes Information System for Policy and Practice	B.I.R.O.	2005–2008
Better Statistics for Better Health for Mothers and their Newborns in Europe	Euro-Peristat	1999 to date
BRidging Information and Data Generation for Evidence-based Health policy and research	BRIDGE	2015–2017
Cancer Control using Population-based Registries and Biobanks	CCPRB	2004–2009
Cancer Registry Based project on Haematologic Malignancies	HAEMACARE	2005–2008
Committee of Nordic Assisted Reproductive Technology and Safety	CoNARTaS	2008 to date
Commonwealth Fund Multinational Comparisons of Health Systems Data	MultiCom	1918 to date
Comparative Effectiveness Research on Psychiatric Hospitalisation by Record Linkage of Large Administrative Data Sets	CEPHOS-LINK	2014–2017
Comparing policy framework, structure, effectiveness and cost-effectiveness of functional and integrated systems of mental health care	COFI	2014–2018
Deepening our understanding of quality improvement in Europe	DUQuE	2009–2014
Developing a Child Cohort Research Strategy for Europe	CHICOS	2010–2013
Diagnosis-Related Groups in Europe - Towards Efficiency and Quality	EuroDRG	2009–2011
Environmental Health Risks in European Birth Cohorts	ENRIECO	2009–2011
EU Public Health Outcome Research and Indicators Collection	EUPHORIC	2004–2008
European Association for Injury Prevention and Safety Promotion	EUROSAFE	2007-nr
EUropean Best Information through Regional Outcomes in Diabetes	EUBIROD	2005 onwards: EUROBIROD project (2008–2012), EUROBIROD Network is ongoing
EUROpean Cancer Registry-based study	EuroCARE	1978 to 2007
European Cardiovascular Indicators Surveillance Set	EUROCISS	2000–2007
European Collaboration for Healthcare Optimization	ECHO	2010–2017
European Community Health Indicators and Monitoring	ECHIM/ECHI	ECHIM JA 2009–2012, 3 ECHI projects 1998–2001, 2001–2004, 2005–2008
European Health Care Outcomes, Performance and Efficiency	EuroHOPE	2010–2014
European Health Data and Evidence Network	EHDEN	2018–2024
European Health Examination Survey	EHES	EHES pilot 2009–2012, ongoing
European Hospital Benchmarking by Outcomes in Acute Coronary Syndrome Processes	EurHOBOP	2009–2012
European Injury Data Base	IDB	2012-nr
European Medical Information Framework	EMIF	2013–2018
European Network for Indicators on Cancer 2006–2009	EUNICE	2005–2007
European Urban Health Indicators System Part 2	EURO-URHIS 2	2009–2013
Extracorporeal life support association	ELSO	1989 to date
Family life courses, intergenerational exchanges and later life health	FAMHEALTH	2013–2018
Global Allergy and Asthma European Network	GA2LEN	2004–2015
Global Burden of Disease	GBD	2007-nr
Health Benefits and Service costs in Europe	HealthBASKET	2004–2007
Health Inequalities Indicators in the Regions of Europe	I2SARE	2008–2010
Improved access to health care data through cross-country comparisons	EuroREACH	2010–2013
Improved methodology for data collection on accidents and disabilities-Integration of European Injury Statistics	INTEGRIS	2008–2011
Individualized cardiovascular disease risk assessment across Europe	EPIC CVD	1990’s − 2009
International Cancer Benchmarking Partnership	ICBP	2009-nr
International Research Project on Financing Quality in Healthcare	InterQuality	2010–2013
Italian nationwide longitudinal population-based study on Diabetic Ketoacidosis at Diagnosis of Type 1 Diabetes	DKA - type 1 diabetes	2016–2018
Joint action on healthy life years	JA EHLEIS	2011–2014
MAnagement of mental health diSorders Through advancEd Technology and seRvices – teleHealth for the MIND	MasterMind	2014–2017
Multinational MONItoring of Trends and Determinants in CArdiovascular Disease	MONICA	1980 onwards. Active period of data collection ended around 2000
Multiple Sclerosis Data Alliance	MSDA	nr
Nordic Welfare dataBASE	NOWBASE	nr
Observational Health Data Sciences and Informatics	OHDSI	2014-nr
Operations management and demand-based approaches to healthcare outcomes and cost-benefits research	MANAGED OUTCOMES	2010–2012
Personalized PREvention of Chronic DIseases consortium	PRECeDI	2015–2018
Pooling of European Data to Harmonise Translational Research in Breast Cancer	ONCOPOOL	2002–2004
Quality and costs of primary care in Europe	EUPrimeCare	2010–2012
Quality and Costs of Primary Care in Europe	QUALICOPC	2010–2013
Registry of Congenital Anomalies	EUROCAT	1979-nr
Research on Children and Adults Born Preterm	RECAP preterm	2017 up to 51 months
Socio-economic inequalities in health and mortality in 16 European cities at the beginning of the twenty-first century	INEQ-CITIES	2009–2012
Surveillance of rare cancers in Europe	RARECARE	2007–2010
Survey of Health, Ageing and Retirement in Europe	SHARE	2004 onwards; wave 8 is ongoing
Tackling Health Inequalities in Europe	EUROTHINE	2004–2007

*nr* not reported

**Fig. 1:**
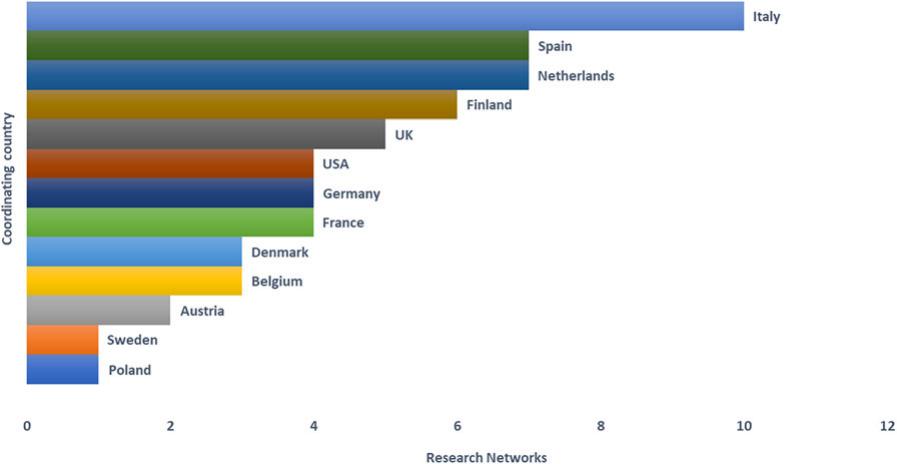
Coordinating countries of the research networks

**Fig. 2:**
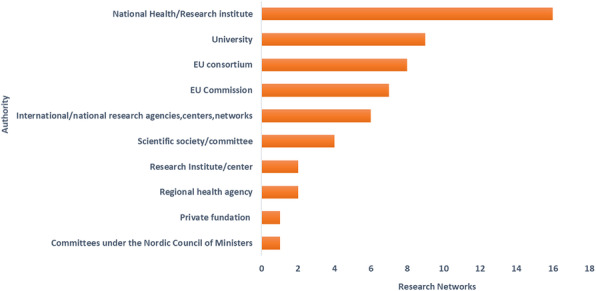
Authorities or responsible organizations of the research networks

A brief description of each RN is reported in Additional file 1. Most RNs were coordinated in Italy (10/57), the Netherlands and Spain (7/57 RNs each) (Fig. 1). National Health or Research Institutes (16/56), Universities (9/56) and the EU consortia (8/56) were the main responsible authorities or organizations of the RNs (Fig. 2). The participating countries of the networks ranged from two countries to the majority or all EU MS. Countries from other geographical regions, such as North and South America (e.g., the USA, Canada, Argentina, Brazil), Africa (e.g., South Africa, Kenya), and Asia (e.g., China, Japan, South Korea) were also part of some RNs. Most networks were representative at national and international level (22/57) or only at international level (16/57), while 14 networks were representative at national, regional and international level.

The RNs used a combination of various health information sources for their research activities, such as administrative data (e.g., hospital discharge records, drug prescription database, mortality register), population-based surveys or interviews, longitudinal or cohort studies, population-based disease registries (e.g., diabetes register, registers of road and workplace injuries), and medical records (e.g., electronic medical charts). The principal area of research for most networks was health monitoring (32/57) and, to a lesser extent, health system performance assessment and monitoring (8/57).

The health topics or diseases considered by the RNs included, but were not limited to, non-communicable diseases (e.g., cardiovascular diseases, diabetes, cancer, mental disorders), unhealthy lifestyles, non-fatal injuries, environmental hazards and urban health, health system performance, healthcare utilization, health inequalities, and health promotion and interventions. According to the main areas of research, various risk factors, high-risk conditions or health behaviors were assessed by the RNs; namely, behavioral, environmental, socio-economic, and disease-specific risk factors. The indicators elaborated from the collected health data included prevalence, incidence, outcome and performance measures, attack rates, injury disability indicators, and more.

Data sharing with other projects or RNs was not in place for most networks (40/49). On the contrary, data sharing was in place for eight RNs (Table 3) and in progress for the Multiple Sclerosis Data Alliance (MSDA) network.

**Table 3:** Data sharing activities of EU research networks

RESEARCH NETWORKS sharing health data	NETWORKS and PROJECTS receiving health data
Best Information through Regional Outcomes: a Shared European Diabetes Information System for Policy and Practice (B.I.R.O.)	EUBIROD
BRidging Information and Data Generation for Evidence-based Health policy and research (BRIDGE)	ECHIM; EHES; ENRIECO; Euro-Peristat, RICHE project, CHICOS; EuroHOPE; EHLEIS
Italian nationwide longitudinal population-based study on Diabetic Ketoacidosis at Diagnosis of Type 1 Diabetes (DKA - type 1 diabetes)	Joint International Project DKA at onset of pediatric type-1 diabetes
European Health Data and Evidence Network (EHDEN)	EHDEN is part of the Innovative Medicines Initiative Big Data for Better Outcome Program (BD4BO)
European Network for Indicators on Cancer (EUNICE)	EuroCARE; EUROCHIP project
European Injury Database (EU-IDB)	EUROSAFE
Multinational MONItoring of Trends and Determinants in CArdiovascular Disease (MONICA)	MORGAM project; euCanSHare project; ENGAGE project; CHANCES consortium; BiomarCARE consortium; AFFECT-EU project
Survey of Health, Ageing and Retirement in Europe (SHARE)	ELSA; U.S Health and Retirement Study

*AFFECT-EU* Digital, risk-based screening for atrial fibrillation in the European community, *BiomarCARE* Biomarker for Cardiovascular Risk Assessment across Europe, *CHANCES* Consortium on Health and Ageing, *CHICOS* Developing a Child Cohort Research Strategy for Europe, *ECHIM* European Community Health Indicators and Monitoring, *EHES* European Health Examination Survey, *EHLEIS* Joint action on healthy life years, *ELSA* English longitudinal study on aging, *ENGAGE* Engage Society for Risk Awareness and Resilience, *ENRIECO* Environmental Health Risks in European Birth Cohorts, *EUBIROD* EUropean Best Information through Regional Outcomes in Diabetes, *euCanSHare* EU-Canada joint infrastructure for next-generation multi-Study Heart research, *EuroCARE* EUROpean Cancer Registry-based study, *EUROCHIP* European Cancer Health Indicator Project, *EUROHOPE* European Health Care Outcomes, Performance and Efficiency, *Euro-Peristat* Better Statistics for Better Health for Mothers and their Newborns in Europe, *EUROSAFE* European Association for Injury Prevention and Safety Promotion, *MORGAM* MOnica Risk, Genetics, Archiving and Monograph, *RICHE* A platform and inventory for child health research in Europe

More than 40 RNs are funded or co-funded by the EU Commission; funds are also provided by Ministries of Health, research councils and various institutes from the participating countries. Five RNs were supported by private foundations (e.g., Bill and Melinda Gates Foundation).

### Quality assurance procedures, data availability, and use of metadata reporting standards

Most RNs (41/56) performed and reported the quality assessment procedures of the collected health data on their website. Quality assessment was not applicable to the Commonwealth Fund Multinational Comparisons of Health Systems Data (MultiCom) which uses data collected and processed by OECD. Out of 49 RNs providing health data on their website, 39 RNs provided only macrodata through reports and/or scientific articles, while only microdata was provided by two networks (Italian nationwide longitudinal population-based study on Diabetic Ketoacidosis at Diagnosis of Type 1 Diabetes-DKA type 1 diabetes and SHARE). Both microdata and macrodata were available from eight RNs (i.e., European Medical Information Framework-EMIF, Registry of Congenital Anomalies-EUROCAT, International Cancer Benchmarking Partnership-ICBP, Surveillance of rare cancers in Europe-RARECARE, MONICA, MSDA, RECAP preterm, and ELSO). Moreover, health data was not reported on the website of eight RNs (i.e., CoNARTaS, EU Public Health Outcome Research and Indicators Collection-EUPHORIC, Developing a Child Cohort Research Strategy for Europe-CHICOS, Environmental Health Risks in European Birth Cohorts-ENRIECO, Best Information through Regional Outcomes: a Shared European Diabetes Information System for Policy and Practice-B.I.R.O., European Health Data and Evidence Network-EHDEN, European Network for Indicators on Cancer-EUNICE, and Cancer Registry Based project on Haematologic Malignancies- HAEMACARE). Further data analysis, such as aggregation or stratification, was possible for 16/47 RNs providing macrodata. The metadata reporting standards used for health data description were specified by 9 out of 49 RNs. These standards were: International Classification of Diseases [6]; International Standard Classification of Occupations 2008 [7]; International Standard Classification of Education, maintained by the United Nations Educational, Scientific and Cultural Organization [8]; Eurostat metadata standards [9]; Observational Medical Outcomes Partnership Common Data Model [10]; and ad-hoc metadata standards.

### Data accessibility

Health data provided by 40 RNs was accessible as macrodata through guidelines, reports and scientific articles; microdata was not provided in open access. A formal request for microdata and/or macrodata access was required by 14 networks, of which three applied a financial charge (i.e., Individualized cardiovascular disease risk assessment across Europe-EPIC CVD, DKA - type 1 diabetes, and ELSO). Data access was usually granted by scientific, ethics, steering or management committees. Overall, data provided by 34 RNs, out of 49, was reusable based on data usage licences (e.g., for a specific project, analysis, period of use, private or public use).

## Discussion

The mapping exercise allowed the identification of EU RNs that were evaluated in terms of collection methods, quality assessment, availability and accessibility of health data. Most RNs used various population health data sources to compile databases covering different health topics. In this light, the data produced by RNs could be considered more reliable and relevant for the development of evidence-based interventions and policy measures compared to single datasets of individual researchers. Accurate and reliable information is the keystone to policy planning and scientific research.

Health information systems collect public health data, analyse and convert data into information for policy-making, ensuring data quality, relevance and timeliness [11]. The status of health information systems is not optimal across EU MS, revealing the scarcity of available, accessible, comparable and reusable health data for research activities and policy making. First of all, health data are not available from a quarter of identified RNs, and only one-third of the networks offer the possibility of further data analysis for specific research purposes. Moreover, microdata are available and accessible upon request from less than 20% of the RNs. Considering data comparability, about 30% of RNs do not provide information on standardized quality assessment procedures of the collected health data, or in some cases, the information is incomplete. In addition, few RNs follow metadata reporting standards for data description. Metadata standards ensure that structured information that defines and describes data is consistent, useful and understood over time [12]. Although about 70% of data provided by the RNs is reusable, the lack of transparency in data collection procedures and analysis observed are critical issues for the health information systems in EU. Infact, adherence to standardized methods ensure the comparability and reusability of high quality research data across time and geographical regions, as well as the integration of various datasets to enhance scientific discoveries [13, 14].

Access to health data, mostly microdata, is granted by various types of committees. This finding is an indicator of the compliance to the General Data Protection Regulation (GDPR) on data protection and privacy in the EU and the European Economic Area (EEA) [15]. Compliance to GDPR enables responsible data sharing while ensuring the appropriate management of personal data within and across EU and associated countries. However, data sharing activities were lacking for over 80% of the RNs, contributing to the paucity of health data that is more evident in times of public health emergencies, such as the COVID-19 pandemic.

Another critical aspect of health information is the fragmentation of health research, which is related to the lifespan of RNs. Indeed, barely 8 of 57 identified RNs are still active. The reasons behind the disruption of research activities are mainly financial resources, relevance and discipline of the projects [Sipido 2020]. In times of financial constraints, resources have to be reallocated at the expense of some research activities. Research based on collaborations across a wide range of disciplines, through an extensive network of researchers, have higher probability to be funded compared to independent scientists or small research groups [1].

The list of RNs included in the mapping exercise was not exhaustive. However, the aim was not to list all existing RNs in EU but to perform a qualitative analysis of identified RNs that could highlight the barriers and facilitating factors related to research networking in EU MS. Moreover, only RNs that are not part of international organizations (e.g., WHO-Europe, OECD, Eurostat) and involved in health data collection for health monitoring and health system performance were considered. This surely limited the number of possible networks that could be included in the study. However, the web-based desk search was enhanced with additional information provided by the partners of the InfAct JA.

## Conclusions

The critical issues related to data quality, availability, accessibility and data sharing underlined by the current findings pose a serious challenge to the scientific advancement and sustainability of the EU information system. Adherence to guidelines and protocols on standardized procedures in data collection and analysis may ensure the comparability and reusability of research results. Moreover, the development of extensive and multidisciplinary RNs could facilitate the optimal allocation of research funds and prevent the fragmentation of research activities.

RNs are essential for the health information system in EU as providers of accurate and reliable health data and have an important role in information exchange between researchers across and outside the EU, and in providing reliable health information for evidence-based health policy decisions. Therefore, RNs could tackle health data and information inequalities by enhancing quality, availability, and accessibility of health data and data sharing across geographical regions.

## Supplementary Information

**Additional file 1.** Brief description of the identified research networks. Description of the research networks.

## Data Availability

The datasets used and/or analysed during the current study are available from the corresponding author on reasonable request.

## Abbreviations

B.I.R.O.: Best Information through Regional Outcomes: a Shared European Diabetes Information System for Policy and Practice
CHICOS: Developing a Child Cohort Research Strategy for Europe
CoNARTaS: Committee of Nordic Assisted Reproductive Technology and Safety
CORDIS: Community Research and Development Information Service
DKA - type 1 diabetes: Italian nationwide longitudinal population-based study on Diabetic Ketoacidosis at Diagnosis of Type 1 Diabetes
ECDC: European Centre for Disease Prevention and Control
ECHO: European Collaboration for Healthcare Optimization
EFSA: European Food Safety Authority
EHES: European Health Examination Survey
EHDEN: European Health Data and Evidence Network
ELSO: Extracorporeal life support association
EMCDDA: European Monitoring Centre for Drugs and Drug Addiction
EMIF: European Medical Information Framework
ENRIECO: Environmental Health Risks in European Birth Cohorts
EPIC CVD: Individualized cardiovascular risk assessment across Europe
EU: European Union
EUBIROD: EUropean Best Information through Regional Outcomes in Diabetes
EUNICE: European Network for Indicators on Cancer 2006–2009
EUPHORIC: EU Public Health Outcome Research and Indicators Collection
EUROCAT: Registry of Congenital Anomalies
EUROCISS: European Cardiovascular Indicators Surveillance Set
Euro-Peristat: Better Statistics for Better Health for Mothers and their Newborns in Europe
Eurostat: European Statistical Office
GDPR: General Data Protection Regulation
HAEMACARE: Cancer Registry Based project on Haematologic Malignancies
ICBP: International Cancer Benchmarking Partnership
InfAct JA: Information for Action - Joint Action on Health Information
MONICA: Multinational MONItoring of Trends and Determinants in CArdiovascular Disease
MS: Member State
MSDA: Multiple Sclerosis Data Alliance
MultiCom: The Commonwealth Fund Multinational Comparisons of Health Systems Data
OECD: Organisation for Economic Co-operation and Development
RARECARE: Surveillance of rare cancers in Europe
RECAP preterm: Research on Children and Adults Born Preterm
RNs: Research Networks
SHARE: Survey of Health, Ageing and Retirement in Europe
WHO: World Health Organization
