# Supplementary material for: Mapping European research networks providing health data: results from the InfAct Joint Action on health information
Source: Arch Public Health. 2022 Jan 10;80:23. doi: 10.1186/s13690-021-00766-2 (PMC8744039; doi:10.1186/s13690-021-00766-2)
Supplement: Supplementary file 1 — Additional file 1. Brief description of the identified research networks. Description of the research networks. [file 13690_2021_766_MOESM1_ESM.docx]

| **RESEARCH NETWORK** | ACRONYM | **DESCRIPTION** |
| --- | --- | --- |
| Best Information through Regional Outcomes: a Shared European Diabetes Information System for Policy and Practice | B.I.R.O. | To build a common European infrastructure for standardized information exchange in diabetes care, for the purpose of monitoring, updating and disseminating evidence on the application and clinical effectiveness of best practice guidelines on a regular basis |
| Better Statistics for Better Health for Mothers and their Newborns in Europe | Euro-Peristat | The objective is to establish a high quality, internationally recognized and sustainable European perinatal information system. |
| BRidging Information and Data Generation for Evidence-based Health policy and research | BRIDGE | To build a system to support interoperability – both technical and social – in large-scale emergency management. The BRIDGE Health project aimed to prepare the transition towards a sustainable and integrated EU health information system for both public health and research purposes |
| Cancer Control using Population-based Registries and Biobanks | CCPRB | To facilitate research linking biobanks and cancer registries |
| Cancer Registry Based project on Haematologic Malignancies | HAEMACARE | This project aims to bridge the gap between clinical research and public health information systems. It will be based on the EUROCARE-3 and EUROPREVAL networks and will profit form the collaboration of EUROCHIP project. Main objective 1. Revision of HMs coding procedures used by cancer registries, ensuring strict adherence to ICD-0 morphology codes, and making them consistent with nosologic categories currently used by clinicians. A panel of haematologists and epidemiologists will be constituted for this purpose. 2. Improve public health use of clinical data. Indicators of clinical activity for HMS by country, will be provided, through integration of data from population cancer registries and clinical networks on HMs. |
| Committee of Nordic Assisted Reproductive Technology and Safety | CoNARTaS | To investigate the health of children born after assisted reproduction (ART), and the safety of ART in four Nordic countries: Denmark, Finland, Norway and Sweden. |
| Commonwealth Fund Multinational Comparisons of Health Systems Data | MultiCom | Data collected by OECD are used to compare healthcare systems and performance on a range of topics (e.g. spending, hospitals, physicians, etc.). Data across eleven industrialized countries are presented: Australia, Canada, France, Germany, the Netherlands, New Zealand, Norway, Sweden, Switzerland, the United Kingdom, and the United States |
| Comparative Effectiveness Research on Psychiatric Hospitalisation by Record Linkage of Large Administrative Data Sets | CEPHOS-LINK | To identify factors related to re-hospitalisations by comparing psychiatric re-hospitalisation rates and identifying their predictors in unselected patient to identify factors related to re-hospitalisations by comparing psychiatric re-hospitalisation rates and identifying their predictors in unselected patient populations from six European countries with different health care systems (Austria, Finland, Italy, Norway, Romania and Slovenia) |
| Comparing policy framework, structure, effectiveness and cost-effectiveness of functional and integrated systems of mental health care | COFI | To conduct a large-scale comparison in countries with different traditions, levels of service provision and funding systems of mental health care (Belgium, Germany, Italy, Poland, United Kingdom). |
| Deepening our understanding of quality improvement in Europe | DUQuE | The main goal of the DUQuE project is to study the effectiveness of quality improvement systems in European hospitals. This has been done by assessing the relationship of organisational quality improvement systems/management and culture, professionals’ involvement, and patient empowerment with the quality of hospital care (including clinical effectiveness, patient safety and patient involvement). |
| Developing a Child Cohort Research Strategy for Europe | CHICOS | Aims to improve child health across Europe by developing an integrated strategy for mother-child cohort research in Europe by evaluating data from existing cohorts, registers and relevant European databases, identifying gaps in knowledge, developing recommendations for targeted research action at the European level. |
| Diagnosis-Related Groups in Europe - Towards Efficiency and Quality | EuroDRG | Analysis of the national DRG-based hospital payment systems by using qualitative and quantitative research methods. |
| Environmental Health Risks in European Birth Cohorts | ENRIECO | Aims to advance knowledge on specific environment and health causal relationships in pregnancy and birth cohorts. |
| EU Public Health Outcome Research and Indicators Collection | EUPHORIC | Aimed at building a consortium of participating countries to cooperate on benchmarking the outcomes of selected health performances and exchange information on quality standards, best practice and effectiveness in public health by developing and maintaining EU networks |
| European Association for Injury Prevention and Safety Promotion | EUROSAFE | Promotes safety of European citizens by enhancing safety of products and services, raises awareness of injury risks at home and in leisure and offers cost effective measures to prevent injuries. |
| EUropean Best Information through Regional Outcomes in Diabetes | EUBIROD | The vision of the EUBIROD Network is to support and facilitate the integration of all diabetes data sources already available, in order to improve all policies against diabetes and to reduce its direct implications across the whole range of non-communicable diseases. An active collaboration of systematic data collection and monitoring of diabetes complications and health outcomes across Europe. The EUBIROD is an informal and voluntary collaboration, which advocates of the rights of the people with diabetes, (non)governmental organizations, scientific societies, associations of health professionals and individual citizens. EUROBIROD network builds on the finished EUROBIROD project 2008-2012). |
| EUROpean Cancer Registry-based study | EuroCARE | Aimed to provide an updated description of cancer survival time trends and differences across European countries, to measure cancer prevalence, and to study patterns of care of cancer patients. |
| European Cardiovascular Indicators Surveillance Set | EUROCISS | Goal was to develop health indicators and recommendations for monitoring the burden and distribution of cardiovascular disease (CVD). Manual of Operations for the implementation of population-based registers of acute myocardial infarction/acute coronary syndrome, stroke and of CVD surveys was the main result. |
| European Collaboration for Healthcare Optimization | ECHO | Aimed at building a common knowledge infrastructure, based on existing datasets. Aims to bring together national hospital databases from several European countries. |
| European Community Health Indicators and Monitoring | ECHIM/ECHI | Aims to create a comparable health information and knowledge system to monitor health at EU level to support policy making. Currently uses over 60 indicators in 5 main themes. These indicators give an overview of health and health systems across Europe. |
| European Health Care Outcomes, Performance and Efficiency | EuroHOPE | To evaluate the performance of European health care systems in terms of outcomes, quality, use of resources and costs. The project focuses on five important disease groups: acute myocardial infarction (AMI), ischemic stroke, hip fracture, breast cancer and very low birth weight and very preterm infants (VLBWI). |
| European Health Data and Evidence Network | EHDEN | Federated data ecosystem in Europe using OMOP common data model. Mission: to provide a new paradigm for the discovery and analysis of health data in Europe, by building a large-scale, federated network of data sources standardized to a common data model |
| European Health Examination Survey | EHES | Population based and objective surveys which provides data on many health indicators to support policy-making, preventive activities and research. Aims to ensure high quality and comparability of health data by standardized procedures. |
| European Hospital Benchmarking by Outcomes in Acute Coronary Syndrome Processes | EurHOBOP | To provide European hospitals with a validated set of statistical functions - including determinants of in-hospital case fatality outcome indicator - to benchmark themselves about the quality of management of myocardial infarction or unstable angina patients and treatments aimed at removing coronary artery occlusion. |
| European Injury Data Base | EU-IDB | It is a surveillance system that provides information on non-fatal unintentional injuries such as home injuries, sports and leisure, workplace and road injuries; in addition to intentional injuries resulting from violence and self-harm. |
| European Medical Information Framework | EMIF | Tackle technical challenges when scaling up real-world health data research; improve access to human health data via providing tools and workflows to discover, assess, access and (re)use human health data |
| European Network for Indicators on Cancer | EUNICE | To establish and operate a network, comprising primary data providers (European Cancer registries) and organizations with experience in coordination, collection, quality control, standardization, processing and dissemination of data, to provide with updated and standardized indicators of cancer. |
| European Urban Health Indicators System Part 2 | EURO-URHIS 2 | The project aims to identify urban health problems, and for the first time to compare health statuses between the different cities in Europe. This is done by developing, testing and validating a set of comparable urban health indicators. The aim is to provide information for policy-makers to prioritise topics in urban health issues, policies and interventions. |
| Extracorporeal life support association | ELSO | An international register; developed a specific dataset in order to help NICE in its assessment of ECMO |
| Family life courses, intergenerational exchanges and later life health | FAMHEALTH | To uncover how family life courses influence health and well-being in later adulthood, whether family related strengths or disadvantages relevant to health offset or compound socio-economic sources of disadvantage, and the extent to which these associations are influenced by societal factors. |
| Global Allergy and Asthma European Network | GA2LEN | International network in allergy and asthma research to bring together institutions and researchers from across EU. The network aims to accelerate the application of research into clinical practice, meet the needs of patients and to help guide policy development. The consortium is leading European research centres specialized in allergic diseases. |
| Global Burden of Disease | GBD | GBD provides a tool to quantify health loss from hundreds of diseases, injuries, and risk factors, so that health systems can be improved and disparities can be eliminated |
| Health Benefits and Service costs in Europe | HealthBASKET | The project developed and tested an innovative approach of cost analysis at the micro-level that allow for international comparisons. |
| Health Inequalities Indicators in the Regions of Europe | I2SARE | To produce a health profile for each region of the European Union, to create a typology of those regions of Europe and a typology of sub regional territories in a selection of countries and regions. Main objective is to assist decisionmakers in developing their health policies, through better understanding in health statuses and health inequalities at regional and subregional levels. |
| Improved access to health care data through cross-country comparisons | EuroREACH | Aims to ensure comparability and harmonization of health data for cross-country research. The project will also provide a toolbox of guidance to researchers, policymakers and other stakeholders interested in cross-country research by: Identifying information sources of patient-level, disease-based data; b) Offering guidance on key data challenges such as data access, linkage and comparability; c) Highlighting gaps in existing data to encourage data collection in underrepresented areas |
| Improved methodology for data collection on accidents and disabilities-Integration of European Injury Statistics | INTEGRIS | To develop and evaluate a data model for the integration of routine and more detailed hospital data on injuries, namely through linking the official HDR (hospital discharge registers) with the EUIDB ( EU Injury database). The goal of the integrated data model would enable hospitals to generate standard injury data with minimal additional efforts. To identify possible risk factors and strengthen injury prevention by understanding statistics from already existing databases. |
| Individualized CVD risk assessment across Europe | EPIC CVD | To provide clinicians and policy-makers with evidence-based options for cost-effective individualised cardiovascular disease (CVD) risk assessment. Encompasses InterAct and EPIC-Heart (projects based on diabetes and coronary heart disease respectively) and include stroke cases |
| International Cancer Benchmarking Partnership | ICBP | The project has demonstrated differences in survival between countries and has suggested some possible causes of these differences, and ruling out possible causes |
| International Research Project on Financing Quality in Healthcare | InterQuality | To investigate the effect of different financing methods and incentives on quality, effectiveness and equity of access to health care in four patient groups affected by: pharmaceutical care, hospital care, outpatient care and integrated care. |
| Italian nationwide longitudinal population-based study on Diabetic Ketoacidosis at Diagnosis of Type 1 Diabetes | DKA - type 1 diabetes | The project provides information on DKA at diagnosis of type 1 diabetes for planning prevention interventions |
| Joint action on healthy life years | JA EHLEIS | To contribute to the first partnership of Innovation Union, which focuses on active and healthy ageing and with the target of increasing by 2 years the average number of healthy life years by 2020. Provides information on various health indicators that are comparable among EU countries and further develops the EHLEIS system to allow rapid access to up-to-date health expectancies. |
| MAnagement of mental health diSorders Through advancEd Technology and seRvices – teleHealth for the MIND | MasterMind | The project created guidelines and pathways for using videoconference for collaborative care and treatment of depression. The guidelines are based on the previous pilot projects. The aim of the project was to use VC (videoconference) in collaborative settings between GPs and specialists. Additionally, the need for the use of it in direct treatment and cCBT (computerised cognitive behavioural therapy) was realised. Main healthcare users can be psychiatric departments, health care organisers, e-health clinics, mental health outpatient services etc. |
| Multinational MONItoring of Trends and Determinants in CArdiovascular Disease | MONICA | The MONICA (Multinational MONItoring of trends and determinants in CArdiovascular disease) Project was established in the early 1980s in many centres around the world to monitor trends in cardiovascular diseases, and to relate these to risk factor changes in the population over a ten year period. It was set up to explain the diverse trends in cardiovascular disease mortality which were observed from the 1970s onwards. There were total of 32 MONICA Collaborating Centres in 21 countries. The total population age 25-64 years monitored was ten million men and women. The ten year data collection was completed in the late 1990s, and the main results were published in the following years. |
| Multiple Sclerosis Data Alliance | MSDA | They work with patient communities and organisations to promote the value of research and the importance of multiple sclerosis data |
| Nordic Welfare dataBASE | NOWBASE | NOWBASE is a shared website for Nordic Medico-Statistical Committee (NOMESCO) and the Nordic Social Statistical Committee (NOSOSCO). Aims to ensure that health and social statistics are comparable in the Nordic Countries. Gathers, presents and distributes data. |
| Observational Health Data Sciences and Informatics | OHDSI | To improve health by empowering a community to collaboratively generate the evidence that promotes better health decisions and better care. |
| Operations management and demand-based approaches to healthcare outcomes and cost-benefits research | MANAGED OUTCOMES | The MANAGED OUTCOMES proposal explores the assumption that healthcare outcomes and costs are affected by the efficiency of service production, the time/location constrained regional structure of healthcare delivery, and the degree to which people are empowered to participate in the co-production of their care. These relationships are insufficiently understood and need to be studied to meet the objectives of the new European health strategy. More optimized health systems should be planned on the grounds of citizens’ expectations for both effective outcomes, and for access and quality of healthcare delivery |
| Personalized PREvention of Chronic DIseases consortium | PRECeDI | Health care is increasingly adopting a more personalised medicine (PM) approach involving individually tailored patient care. The project aims to foster collaboration on PM research and training with attention to prevention of chronic diseases. A set of recommendations in research and consortium was carried out to policy-makers, scientists and health care industry. |
| Pooling of European Data to Harmonise Translational Research in Breast Cancer | ONCOPOOL | The proposal is to obtain a large Data Set of Breast cancers, from Breast Units representative of the presentation of the disease in Europe. No such large tumour set, with carefully compiled and checked long-term clinical follow-up and with standardised histological and other measurements of tumour factors, exists in Europe. The Data Set will provide a critical mass, invaluable in translational research for relating tumour factors to clinical outcomes. The Consortium for the project provides a European Network of Excellence in Breast Cancer. At the conclusion of the project this will be expanded by other Units. In turn this will provide a huge Data Set with associated Quality Assurance of the data. |
| Quality and costs of primary care in Europe | EUPrimeCare | Aimed to develop a framework to analyse Primary Care across Europe, to assess and compare Primary Care models in terms of quality and identifying costs and to provide recommendations. |
| Quality and Costs of Primary Care in Europe | QUALICOPC | Aims to analyse and compare how primary health care systems in 34 countries perform in terms of quality, costs and equity |
| Registry of Congenital Anomalies | EUROCAT | Gathers, validates, analyses and disseminates data on Congenital Anomalies and its determinants at country level and regional level in EU Countries. Promotes data use in collaborative research projects |
| Research on Children and Adults Born Preterm | RECAP preterm | To improve health, development and quality of life of very preterm or very low-birth-weight children and adults by combining European cohort studies |
| Socio-economic inequalities in health and mortality in 16 European cities at the beginning of the 21st century | INEQ-CITIES | To identify socio-economic inequalities in health and mortality in Europe and to examine urban health policies developed to tackle such inequalities in health. |
| Surveillance of rare cancers in Europe | RARECARE | Aim is to provide an operational definition of “rare cancer” and a list of cancers meeting that definition |
| Survey of Health, Ageing and Retirement in Europe | SHARE | Multidisciplinary and cross-national panel database of microdata on health, socio-economic status and social and family networks of individuals aged 50 or older |
| Tackling Health Inequalities in Europe | EUROTHINE | The Eurothine project is a large international project aimed to improve the description of health inequalities in Europe and to enhance the evidence-base for policies to reduce inequalities in health. Its two principal objectives were: 1) to prepare international overviews that provide bench-marking data on inequalities in mortality, morbidity and health determinants to participating countries; 2) to assess evidence on the effectiveness of policies and interventions to tackle health inequalities, and to make recommendations on strategies for reducing health inequalities in participating countries |
